# Anatomical variations of the recurrent laryngeal nerve in Chinese patients: a prospective study of 2,404 patients

**DOI:** 10.1038/srep25475

**Published:** 2016-05-05

**Authors:** Tanglei Shao, Weihua Qiu, Weiping Yang

**Affiliations:** 1 Department of General Surgery, Ruijin Hospital affiliated to Shanghai Jiaotong University School of Medicine, Shanghai 200025, China

## Abstract

The recurrent laryngeal nerve (RLN) shows some anatomical variations that can potentially compromise the safety of thyroid surgery. The purpose of this prospective study was to identify the anatomical variations of the RLN in Chinese patients undergoing thyroid surgery. Between January 2007 and December 2013, 2,404 Chinese patients were hospitalized for thyroid surgery with dissecting of the RLN unilaterally or bilaterally. The patients consisted of 510 men and 1,894 women, with a median age of 45.0 years. Overall 3,275 RLNs, including 1,576 left- and 1,699 right-side nerves, were dissected. The anatomical variations were identified in 690 RLNs, including 305 left- and 385 right-side nerves. We identified as many as seven RLN anatomical variations in Chinese patients. These findings indicate that anatomical variations of the RLN are common, and the identification of these anatomical variations of the RLN can help to minimize the risk of post-operative RLN paralysis.

The recurrent laryngeal nerve (RLN) is a branch of the vagus nerve, which carries motor, sensory and parasympathetic fibers to the larynx. The RLN is consistently present superior to the inferior thyroid artery before it ascends behind the inferior constrictor to the nerve’s entry point into the larynx. This relationship is a major landmark for its identification during thyroid surgery. However, anatomical variations of the RLN represent a well-known risk factor of RLN injury in thyroid surgery. Injury to the RLN can cause RLN paralysis with symptoms ranging from almost undetectable hoarseness in unilateral lesions to stridor and acute airway obstruction in bilateral damage^1^,^2^. Transient post-operative RLN paralysis occurs in approximately 3–8% of cases and permanent paralysis in 0.3–3% of cases^3^,^4^,^5^. Hence, it is of importance to identify the anatomical variants of the RLN in order to preserve the nerve and its function during surgery.

Although recent monitoring advances have allowed intraoperative neuromonitoring to reduce the incidence of RLN injury, visual identification of the RLN remains the gold standard for RLN injury prevention^6^. Fully understanding the anatomical variations of the RLN may help surgeons dissect the RLN in a safe, fast, and correct way. Therefore, it is vital to determine the anatomical position of the RLN in thyroid surgery.

In this study, we identified the anatomical variations of the RLN in a large population of Chinese patients undergoing thyroid surgery to explore RLN variations and compare with those reported in Western patients.

## Patients and Methods

### Patients

The study was approved by the institutional review board of Ruijin Hospital and performed in accordance with the ethical principles of the Declaration of Helsinki (Ruijin LL-14-2006). Written informed consent was obtained from each participant before study entry. This prospective study included all patients consecutively hospitalized for elective thyroidectomy due to benign or malignant thyroid diseases between January 2007 and December 2013. The inclusion criteria were as follows: age of 18 years or greater; diagnosis of a surgically indicated thyroid disease, namely, thyroid cancer suspected on ultrasound or diagnosed via fine-needle aspiration biopsy, undetermined single or multiple solid nodules at the maximum size over 30 mm, goiter of any nature compressing the trachea, or retrosternal goiter. The exclusion criteria were as follows: pregnancy, lactation, a history of previous neck surgery or radiation, pre-existing RLN impairment on preoperative laryngoscopy, complicating serious cardiopulmonary, hepatorenal and coagulation disorder.

### Intraoperative identification of the RLN

Routine hematologic, clinical biochemistry, and coagulation function assays as well as electrocardiography and a pulmonary ventilation function test were performed for all patients. Preoperative laryngoscopy excluded the presence of any clinically significant vocal cord impairment in all included patients. All operations were performed by the same assigned surgical team led by a board-certified general surgeon (TS), including resident surgeons, anesthesiologists, radiologists, clinical pathologists, otolaryngologists, surgical nurses, and research staff. The patients were anesthetized under general endotracheal anesthesia. A standard thyroid collar incision of 6–8 cm was made 2–3 cm above the sternal notch and clavicles and extended right over the sternocleidomastoid muscle. The superior pedicle was ligated, and Berry’s ligament was divided. The dissection began by transecting the isthmus of the thyroid gland following isolation of the strap muscles from the thyroid gland, after which the middle thyroid vein was taken and the superior pole was mobilized. Branches of the superior pole were isolated, ligated, and transected successively. The RLN was identified in a triangle bound laterally by the common carotid artery, medially by the trachea, and superiorly by the thyroid lobe. The exposure was initiated at the entry of RLN into the larynx with the whole portion dissected along with the fibers of the RLN. For those that were tightly adhered, the anterior borders of the sternocleidomastoid muscles were mobilized, and the RLN was identified by a “back door” approach after medial reflection of the sternothyroid muscle. The entire course of the RLN was traced to the level of entry into the larynx under the cricothyroid muscle along the trachea-esophageal groove, and the nerves were dissected from fat and connective tissue. The left-side RLN was identified along the left-side tracheoesophageal groove and in proximity to the middle or lower pole. The right-side RLN was exposed medially to the common carotid artery along the right-side tracheoesophageal groove and in proximity to the middle pole. The RLN extending the entire length of the neck was meticulously dissected for identification and characterization of RLN anatomical variations using macroscopy. Operative data obtained prospectively included the location of the nerve, the number of branches, the distance in millimeters from the inferior border of the cricothyroid to the point of bifurcation, and the distance from the point of bifurcation to the entry into the larynx.

### Statistical analysis

The statistical software package SPSS 16.0 (SPSS Inc., Chicago, IL, USA) was used for statistical analysis. All categorical data were expressed as n (%) and compared using the Chi-Square test. A value *P* < 0.05 was considered statistically significant difference.

## Results

Overall, 2,404 patients undergoing thyroid surgery as scheduled were included for analysis. This prospective study population consisted of 510 men and 1,894 women, with a median age of 45.0 years (range, 12–80 years). A total of 3,275 RLNs, including 1,576 left- and 1,699 right-side nerves, were dissected, with unilateral exposure in 1,533 patients and bilateral exposure in 871 patients.

The identified anatomical variations of the RLN in Chinese patients are outlined in Table 1. Overall, 690 (21.1%) RLNs exhibited anatomical variation, involving 305 (19.4%) left- and 385 (22.7%) right-side RLNs, with no significant difference in laterality (*P* = 0.070).

**Figure 1:**
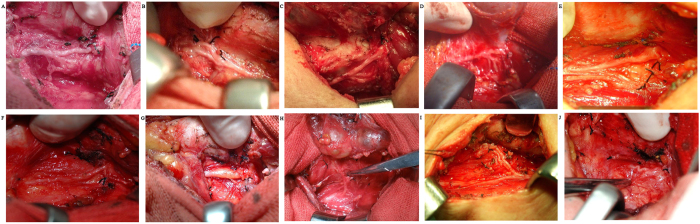
Anatomic variations of recurrent larynx nerve in Chinese patients, including diverging (**A**) and converging (**B**) entry of bifurcation, trifurcation (**C**), quadrifurcation (**D**), fan-shaped divergence at the entry into the larynx (**E**), laryngeal entry >5 mm distant to the cricothyroid joint (**F**), degenerated recurrent larynx nerve in the elderly (**G**), non-recurrent larynx nerve (**H**), intracranial branch (**I**) and tortuous upward-leading RLN (**J**).

These variations were classified according to descending frequency as variations in extralaryngeal structures. The type I comprised the RLN that walked with extralaryngeal branches before entering the larynx, including bifurcation (diverging entry and converging entry) (Fig. 1A,B), trifurcation (Fig. 1C), and quadrifurcation (Fig. 1D). Type II was a fan-shaped RLN before entering the larynx (Fig. 1E). Type III was characterized by entry of the nerve trunk into the larynx far away from the cricothyroid joint (Fig. 1F). The distance from the entry to the back of cricothyroid joints was greater than 5 mm. Type IV was a degenerated RLN observed in elderly patients over the age of 60 years only (Fig. 1G). The degenerative RLN thickened and showed adipopexis. In type V, instead of originating from the aortic arch, the nerve passed from the vagus nerve in the neck and entered the larynx directly, namely the nonrecurrent laryngeal nerve (NRLN) (Fig. 1H). Type VI was characterized by entry of the upward branch into the cranium before entry into the larynx (Fig. 1I). Type VII was a tortuous upward-leading RLN (Fig. 1J).

The type I variation was identified significantly more frequently in the left-side RLN (*P* = 0.022). The type II variation was observed more frequently in the right-side RLN (*P *< 0.001), whereas the type III variation occurred more frequently in the left-side RLN (*P *= 0.029). The type IV variation occurred in the elderly patients and manifested as obvious adipose deposition and thickening of the RLN without a significant difference in laterality (*P* = 0.260). The type V variation was observed in right-side RLN only. The type VI variation was also observed in right-side RLN only, and the type VII variation showed no significant difference in laterality (*P* = 0.570).

## Discussion

The present study is the first large-scale prospective study evaluating the anatomical variations of the RLN in a Chinese population. Previous studies have reported three types of anatomical variations of the RLN, including variations in extralaryngeal divergence (type I), fan-shaped divergence before the entry to the larynx (type II), and the rarely encountered NRLN (type V)^7^,^8^,^9^,^10^. In addition to these variants that were identified in Western and Middle Eastern populations, we identified four other anatomical variants of the RLN in Chinese thyroid disease patients. Among these four variants, the type III variation, in which the RLN with a laryngeal entry located >5 mm away from the cricothyroid joint, was reported previously by our group^11^. The other three variants, including the presence of a degenerated RLN in the elderly (type IV), intracranial branching (type VI), and a tortuous upward-leading RLN (type VII), are identified in Chinese patients for the first time in the present study.

The extralaryngeal branching of the RLN is a common anatomical variation encountered in thyroid surgery and represents a source of increased surgical morbidity. In some reports, extralaryngeal branching of the RLN occurs with up to 36% with the branching of the RLN into two or more divisions on one or both sides^12^. Other researchers have found similar results regarding the frequency of the RLN bifurcation. A large series showed that approximately 72% of RLNs branch before entering the larynx. In this study, the rate of bifurcation, trifurcation, and quadrifurcation of the RLN was 11.5%, which is lower than the percentages reported in other investigations. Despite its low prevalence, the NRLN is an important anatomical variation to be aware of, not only during thyroidectomy but also during other surgical procedures as it can result in vocal cord paralysis if ligated accidentally. This study examined the incidence of NRLN and found that it was present only in 0.5% of cases.

The RLN runs a different course on each side: on the left, the RLN originates from the vagus nerve at the aortic arch, passes below the ligamentum arteriosum, hooks around the aortic arch, returns into the neck within the transoesophageal groove, and then enters the larynx posterior to the inferior constrictor. On the right, it originates at the level of the first part of the subclavian artery and loops below the transoesophageal groove before entering the larynx. In terms of laterality of the variation, studies have reported varying arrangements^13^,^14^. In the present study, we observed a significantly higher rate of anatomical variation of the right RLN compared with the left RLN. The right RLN tends to be more variable in its position, both in relation to the inferior thyroid artery and also in being more anterolateral than the left RLN, which almost always tends to lie in the trachea-oesophageal groove. This could be explained by the embryological origin of the RLN.

There are some limitations to the present study. First, we selected patients undergoing thyroid surgery, and thus, the ratio of male to female patients was 1:3. Therefore, we observed the anatomical variations of the RLN in more female Chinese individuals. It is necessary to identify the anatomical variations of the RLN among more male individuals in future studies. Second, the sample size was relatively small for some specific RLN anatomical variations, such as types IV–VII, despite the large number of total patients included in the current study. Third, information regarding the increased risk of injury associated with the anatomical variation of the RLN is sparse. Future multi-institutional studies elucidating whether patient populations with more frequent anatomical variations of the RLN experienced increased rates of RLN injury are needed to clarify this point.

## Additional Information

**How to cite this article**: Shao, T. *et al*. Anatomic variants of the recurrent laryngeal nerve in Chinese patients: a prospective study of 2,404 patients. *Sci. Rep*. **6**, 25475; doi: 10.1038/srep25475 (2016).

## Figures and Tables

**Table 1:** Anatomical variations of the recurrent larynx nerve.

Anatomical variation	n (%)	Laterality left/right n (%)/n (%)	*P*
Type I (extralaryngeal divergence)	376 (11.5)	205 (13.0)/171 (10.1)	0.022
Type II (fan-shaped divergence)	234 (7.2)	61 (3.9)/173 (10.2)	<0.001
Type III (laryngeal entry >5 mm distant to the cricothyroid joint)	37 (1.1)	25 (1.6)/12 (0.7)	0.029
Type IV (degenerated recurrent larynx nerve in the elderly)	17 (0.5)	11 (0.7)/6 (0.4)	0.260
Type V (non- recurrent larynx nerve)	15 (0.5)	0 (0.0)/15 (0.9)	–
Type VI (intracranial branch)	7 (0.2)	0 (0.0)/7 (0.4)	–
Type VII (tortuous upward-leading recurrent larynx nerve)	4 (0.1)	3 (0.2)/1 (0.06)	0.570
